# Transplanted human iPSC-derived vascular endothelial cells promote functional recovery by recruitment of regulatory T cells to ischemic white matter in the brain

**DOI:** 10.1186/s12974-023-02694-0

**Published:** 2023-01-17

**Authors:** Bin Xu, Hiroya Shimauchi-Ohtaki, Yuhei Yoshimoto, Tetsushi Sadakata, Yasuki Ishizaki

**Affiliations:** 1 Department of Molecular and Cellular Neurobiology, Gunma University Graduate School of Medicine, Maebashi, 3-39-22 Showa-Machi, Maebashi, Gunma 371-8511 Japan; 2 Department of Neurosurgery, Gunma University Graduate School of Medicine, Maebashi, Gunma Japan; 3 Education and Research Support Center, Gunma University Graduate School of Medicine, Maebashi, Gunma Japan; 4 Department of Pathology, The First Affiliated Hospital, Zhejiang University School of Medicine, Hangzhou, Zhejiang China

**Keywords:** Ischemic stroke, Demyelination, Cell transplantation, iPS cells, Endothelial cells, Neuroinflammation, Regulatory T cells, Microglia

## Abstract

**Background:**

Ischemic stroke in white matter of the brain induces not only demyelination, but also neuroinflammation. Peripheral T lymphocytes, especially regulatory T cells (Tregs), are known to infiltrate into ischemic brain and play a crucial role in modulation of inflammatory response there. We previously reported that transplantation of vascular endothelial cells generated from human induced pluripotent stem cells (iVECs) ameliorated white matter infarct. The aim of this study is to investigate contribution of the immune system, especially Tregs, to the mechanism whereby iVEC transplantation ameliorates white matter infarct.

**Methods:**

iVECs and human Tregs were transplanted into the site of white matter lesion seven days after induction of ischemia. The egress of T lymphocytes from lymph nodes was sequestered by treating the animals with fingolimod (FTY720). The infarct size was evaluated by magnetic resonance imaging. Immunohistochemistry was performed to detect the activated microglia and macrophages, T cells, Tregs, and oligodendrocyte lineage cells. Remyelination was examined by Luxol fast blue staining.

**Results:**

iVEC transplantation reduced ED-1^+^ inflammatory cells and CD4^+^ T cells, while increased Tregs in the white matter infarct. Treatment of the animals with FTY720 suppressed neuroinflammation and reduced the number of both CD4^+^ T cells and Tregs in the lesion, suggesting the importance of infiltration of these peripheral immune cells into the lesion in aggravation of neuroinflammation. Suppression of neuroinflammation by FTY720 per se, however, did not promote remyelination in the infarct. FTY720 treatment negated the increase in the number of Tregs by iVEC transplantation in the infarct, and attenuated remyelination promoted by transplanted iVECs, while it did not affect the number of oligodendrocyte lineage cells increased by iVEC transplantation. Transplantation of Tregs together with iVECs into FTY720-treated ischemic white matter did not affect the number of oligodendrocyte lineage cells, while it remarkably promoted myelin regeneration.

**Conclusions:**

iVEC transplantation suppresses neuroinflammation, but suppression of neuroinflammation per se does not promote remyelination. Recruitment of Tregs by transplanted iVECs contributes significantly to promotion of remyelination in the injured white matter.

**Supplementary Information:**

The online version contains supplementary material available at 10.1186/s12974-023-02694-0.

## Background

Stroke is one of the main causes of disability and even death throughout the world, but there is no effective treatment so far [1–3]. Ischemic stroke is the major type of stroke, accounting for about 80% of the whole stroke [4]. As a vulnerable site of ischemia, white matter infarct is an important factor leading to sensorimotor and cognitive dysfunction [5]. Recent studies have shown that neuroinflammation plays an important role in the progress of cerebral ischemic injury [6]. After ischemic stroke in white matter, microglia and macrophages are activated, then release a large number of pro-inflammatory cytokines such as tumor necrosis factor α (TNF-α) and interleukin 1β (IL-1 β) and produce matrix metalloproteinases (MMPs) such as MMP-9 that disrupt the integrity of the blood–brain barrier [7], thereby promoting infiltration of immune cells such as T lymphocytes. These infiltrating immune cells further amplify the neuroinflammatory response, promote demyelination of the white matter area, and ultimately contribute to exacerbation of infarction [8]. In addition, a persistent pro-inflammatory microenvironment after ischemic stroke is considered a potential mechanism that hinders remyelination and recovery of white matter [9]. Therefore, suppressing progression of neuroinflammation seems a good strategy for the treatment of white matter ischemia.

Our previous reports showed transplantation of rat brain microvascular endothelial cells and human induced pluripotent stem cell (iPSC)-derived vascular endothelial cells (iVECs) improves locomotor deficit, reduces infarct area, promotes remyelination, and suppresses neuroinflammation [10, 11]. We also showed that transplantation of vascular endothelial cells into the infarct area promotes increase of oligodendrocyte precursor cells (OPCs) in the region [12]. We further showed that extracellular vesicles (EVs) secreted from vascular endothelial cells promote survival, proliferation, and motility of cultured OPCs [13], and that fibronectin on the surface of EVs contributes to their internalization into OPCs and promotes OPC survival and proliferation [14]. The mechanism of how transplanted vascular endothelial cells suppress neuroinflammation, however, remains unclear. Elucidation of the mechanism may prove useful for establishment of effective therapeutic strategy for prevention and treatment of ischemic stroke.

In the present study, we aimed to investigate contribution of the immune system, especially regulatory T cells (Tregs), to the mechanism whereby iVEC transplantation ameliorates white matter infarct and promotes functional recovery. As the responses of T cells following stroke have been well documented in previous studies [15–18], we focused on the responses of immune cells after iVEC transplantation in this study.

## Materials and methods

### Animals

Eight-week-old male Sprague–Dawley (SD) rats (250–270 g weight) were sourced from SLC (Shizuoka, Japan). All rats were housed in animal facilities with a total of two animals per cage in a temperature-controlled room with a 12-h light/dark cycle and were fed standard diet ad libitum with free access to the water. The rats were adaptively fed for 3–5 days before the initiation of the study. Rats were randomly assigned to individual groups. All efforts were made to use only the number of animals necessary to produce reliable results.

### iPSC culture and iVEC differentiation

Human IMR90-4 iPSC line was obtained from WiCell and maintained on growth factor-reduced Matrigel (BD Bioscience, Cat# 356230) coated 6-well plate (Corning) in mTeSR1 culture medium (STEMCELL Technologies, Cat# ST-85850). 4–6 days after daily culture medium changes, iPSCs were routinely passaged in colony format using Versene (Gibco, Cat# 15040066) at a typical ratio 1:10. All iPSCs differentiated into endothelial cells were used between passages 34 to 44 for preventing chromosomal instability and differentiation bias induced by stress associated with passaging methods and culture conditions.

iVEC differentiation from human iPSCs was performed according to previously described [11]. Briefly, iPSCs were singularized with Accutase (Millipore, Cat# SCR005) and seeded on growth factor-reduced Matrigel-coated 6-well plate at a density of 10,000 cells/cm^2^. 10 μM Y27632 (ROCK inhibitor; Fujifilm Wako Pure Chemicals, Cat# 036-24023) was dissolved in mTeSR1 culture medium at the first 24 h for promoting cell attachment and keeping a spread and mesenchymal-like morphology. It was withdrawn by replacing the culture medium after 24 h. When iPSCs grew into a suitable density (30,000 cells/cm^2^ is optimal; 25,000–40,000 cells/cm^2^ is acceptable), the differentiation was initiated by replacing the culture medium to unconditioned medium (UM: DMEM/F12 (1:1) (Gibco, Cat# 11330032) containing 20% knock-out serum replacement (KOSR, Gibco, Cat# 10828010), 1% MEM non-essential amino acids (Gibco, Cat# 11,140,050), 0.5% GlutaMAX (Gibco, Cat# 35050-061), and 0.1 mM β-mercaptoethanol (NACALAI TESQUE, Cat# 21417-52). Culture medium was replaced daily with fresh UM. After 6 days of incubation, the UM was replaced with human endothelial cell medium constituted by human endothelial serum-free medium (hESFM, Gibco, Cat# 11111044) supplemented with 1% platelet-poor human plasma derived serum (PDS, Sigma, Cat# P2918), 20 ng/ml human basic fibroblast growth factor (R&D Systems, Cat# 233-FB) and 10 µM retinoic acid (Fujifilm Wako Pure Chemicals, Cat# 188-01113). After 48 h incubation, the differentiated cells were dissociated with Accutase and plated on tissue culture dishes coated with collagen IV (80 µg/ml; Sigma, Cat# C5533) and fibronectin (20 µg/ml; Sigma, Cat# F2518). One day after subculture, the cells were washed twice with phosphate-buffered saline (PBS) containing calcium and magnesium (Gibco, Cat# 14040133) to remove non-attached and dead cells. Finally, these differentiated cells were cultured in hESFM supplemented with 1% PDS.

### Human Tregs isolation, expansion, and detection

Human whole blood was collected from healthy donors (demographic data of donors are listed in Table 1) into SepMate™ tubes (STEMCELL, Cat# ST-86415). After treating with RosetteSep™ human total lymphocyte enrichment cocktail (STEMCELL, Cat# ST-15223), the whole blood was diluted by sterile PBS. Peripheral blood mononuclear cells (PBMCs) were isolated from the diluted blood samples by density gradient centrifugation with Lymphopre™ medium (STEMCELL, Cat# ST-07801). Then Tregs were isolated from PBMCs by magnetic-activated cell sorting (MACS) with a CD4^+^CD25^+^ regulatory T cell isolation kit (Miltenyi Biotec, Cat# 130-091-301) according to the manufacturer’s protocol. Briefly, non-CD4^+^ cells were magnetically labeled with an antibody cocktail and then were depleted by negative selection over an autoMACS column (Miltenyi Biotec, Cat# 130-021-101), which was placed in the magnetic field of an autoMACS Pro Separator (Miltenyi Biotec). Then these cells were labeled with CD25 MicroBeads and performed positive selection. The positive selection step was separated twice consecutively to increase the purity of CD4^+^CD25^+^ Tregs.

**Table 1:** The population characteristics of the healthy PBMCs donors

Donor	Sex	Age	Auto-immune diseases	Immune deficiencies
1	Male	32	None	None
2	Male	67	None	None
3	Female	31	None	None

The isolated Tregs were expanded using a Treg Expansion kit (Miltenyi Biotec, Cat# 130–095-345). Human Tregs were cultured in TexMACS™ medium (Miltenyi Biotec, Cat# 130–097-196) supplemented with 500 U/mL human rIL-2 (Miltenyi Biotec, Cat# 130–097-745), 5% human AB serum (VERITAS, Cat# ACC-HAB-7102MH) and 0.01 mM β-mercaptoethanol. Tregs were stimulated by MACSiBead™ Particles preloaded with CD3 and CD28 antibodies. After 14 days of Treg expansion, the MACSiBead™ Particles were removed by MACSiMAG Separator (Miltenyi Biotec, Cat# 130–092-168).

The expanded Tregs were detected by flow cytometry using a human Treg Detection Kit (Miltenyi Biotec, Cat# 130–122-994) according to the manufacturer’s instructions. Briefly, Tregs were labeled with surface markers such as CD4 and CD25. Then these cells were intracellular stained with Foxp3 antibody after fixation and permeabilization. The cells were analyzed using a FACSAria™ II flow cytometer (BD Bioscience). CD4^+^ T cells were gated as P1. From P1-gated population, Tregs were gated as CD25^+^Foxp3^+^ cells.

### Stereotactic ET-1 injection, cell transplantation and FTY720 administration

White matter infarct model was established by endothelin-1 (ET-1) injection into brain internal capsule (IC) as previously described with some modifications [11, 19]. Rats were placed in an animal stereotactic frame after anesthesia with isoflurane. A homeothermic pad was used to maintain the body temperature. The posterior limb of the left IC was the target for injection (2.0 mm posterior and 6.6 mm lateral from the bregma, 4.6 mm depth from the brain surface). A 33-gauge needle was angled away from the injection site at 30° to minimize the mechanical damage to primary motor, sensorimotor and hippocampal structures, and avoid the leakage of ET-1 into lateral ventricle. 1 µl of ET-1 solution (100 pmol/µl, Peptide Institute, Cat# 4198-s) with sterile PBS was injected into the targeted IC at a constant flow rate (0.2 µl/min) via a 10-µl syringe (Hamilton) connected to a micropump. After ET-1 injection, the needle was left in place for 10 min to avoid backflow, and then it was slowly removed from the brain. Rats were allowed to recover for 2 h in a 28 °C room before returning to the home feeding room to minimize animal suffering.

One week after ET-1 injection, when the model of white matter ischemia was established, 2 µl of the iVEC suspension suspended in HBSS containing 0.2% BSA (0.2% BSA/HBSS) at a density of 50,000 cells/µl, or 2 µl of iVEC and Treg (1:1) suspension suspended in 0.2% BSA/HBSS at a density of 50,000 cells/µl was injected into each rat brain using the coordinates used for ET-1 injection. As control, 2 µl of 0.2% BSA/HBSS was injected instead of iVECs or Tregs. Rats received intraperitoneal administration of fingolimod (FTY720, 1 mg/kg; Sigma-Aldrich, Cat# SML0700) daily from day 7 to day 14.

### Magnetic resonance imaging

Seven days after ET-1 injection, magnetic resonance (MR) imaging was performed before cell transplantation or vehicle injection by a small animal 1-T benchtop MR scanner (Icon; Bruker Biospin, Germany). Rats were anesthetized with 5% isoflurane and maintained with 1.5% isoflurane. The body temperature was maintained at 37 °C, and respiration rates were monitored throughout the procedure. T2-weighted image was captured to determine the precise lesion location: rapid- acquisition relaxation enhancement factor 5, repetition time 2500 ms, echo time 60 ms with in-plane resolution of 266 × 266 µm^2^, thickness 1250 µm, and 5 slices.

### Cryosection

Rats were deeply anesthetized to reduce the suffering with isoflurane and perfused transcardially with 4% paraformaldehyde (PFA) in PBS containing 10 U/ml heparin. The brain tissue was immediately dissected and post-fixed for 1 h in the same fixative at 4 °C. After that, the tissue of brain was cryoprotected in 20% sucrose at 4 °C for 2 days and then in 30% sucrose until equilibrated. Frozen coronal sections (20 µm in thickness, 120 serial slices per brain) with Tissue-Tek O.C.T. Compound (Sakura Finetek) were cut using a cryostat (CM3050S; Leica Biosystems, Germany), mounted on pre-coated glass slides (Matsunami, Japan), and placed at room temperature for 1 h to air dry. Then the sections were stored at -80 °C until staining.

### Immunohistochemical staining

Brain sections were incubated with 3% BSA in PBS containing 0.3% Triton X-100 for 30 min. Antigen retrieval was performed at 120 °C for 3 min (for CD4 and Fxop3 staining) and 20 min (for Olig2 staining) in trisodium citrate buffer (10 mM, pH 6.0) before blocking nonspecific binding sites. Then the samples were incubated with primary antibodies including mouse monoclonal antibodies directed against ED-1 (1:400, Bio-Rad, Cat# MCA341R), CD 4 (1:500, Bio-Rad, Cat# MCA372G) and STEM121 (1:500, Takara Bio, Cat# Y40410), a rat monoclonal antibody directed against Foxp3 (1:500, Invitrogen, Cat# 13-5773-82) and a rabbit polyclonal antibody directed against Olig2 (1:200, IBL, Cat# 18953) at 4 °C overnight. For ED-1, STEM121, and Olig2 staining, after washing with PBS, the sections were incubated with fluorophore-conjugated secondary antibodies for 2 h at room temperature. For CD 4 and Foxp3 detection, the samples were stained by TSA system (Perkin Elmer) to amplify the positive tyramide signal. Nuclei were stained with Hoechst 33342 (1µg/ml, Sigma, Cat# B2261) for 1 h at room temperature. Following rinsing by PBS, the sections were mounted with coverslips using Vectashield mounting medium. All stained brain sections were observed using a fluorescent microscope (Axioplan2, Zeiss) with Cooled CCD Camera (DP73, Olympus) or a fluorescence microscope (BZ-X710, Keyence, Japan).

### Luxol fast blue staining

Myelin was stained with Luxol fast blue (LFB) according to our previously method [11]. To calculate the volume of the IC, 12 slices were stained across the injured zone (distance between slices: 200 µm) and the left and right IC areas were measured in each slice. Sections were visualized using a fluorescence microscope (BZ-X710, Keyence, Japan). The boundary of intact IC was drawn manually and the area of the IC was measured by the software. The average IC area between two adjacent sections was multiplied by the thickness and distance between them. Then the volume of the IC was summated. The IC measurement was the percentage of the ipsilateral IC volume to the contralateral IC volume.

### Statistical analysis

All values are shown as mean ± standard error of the mean (SEM). A two-tailed Student’s t-test was performed to compare the differences between two groups. One-way analyses of variance (ANOVA) followed by the post hoc Tukey–Kramer test was assessed to compare the means among three or more groups. All statistical analysis was performed, and then quantified into graphs by using GraphPad Prism 9.0 (GraphPad Prism Software). Statistical significance was inferred at P-values of less than 0.05.

## Results

### Transplantation of iVECs suppressed neuroinflammation in white matter infarct

**Fig. 1:**
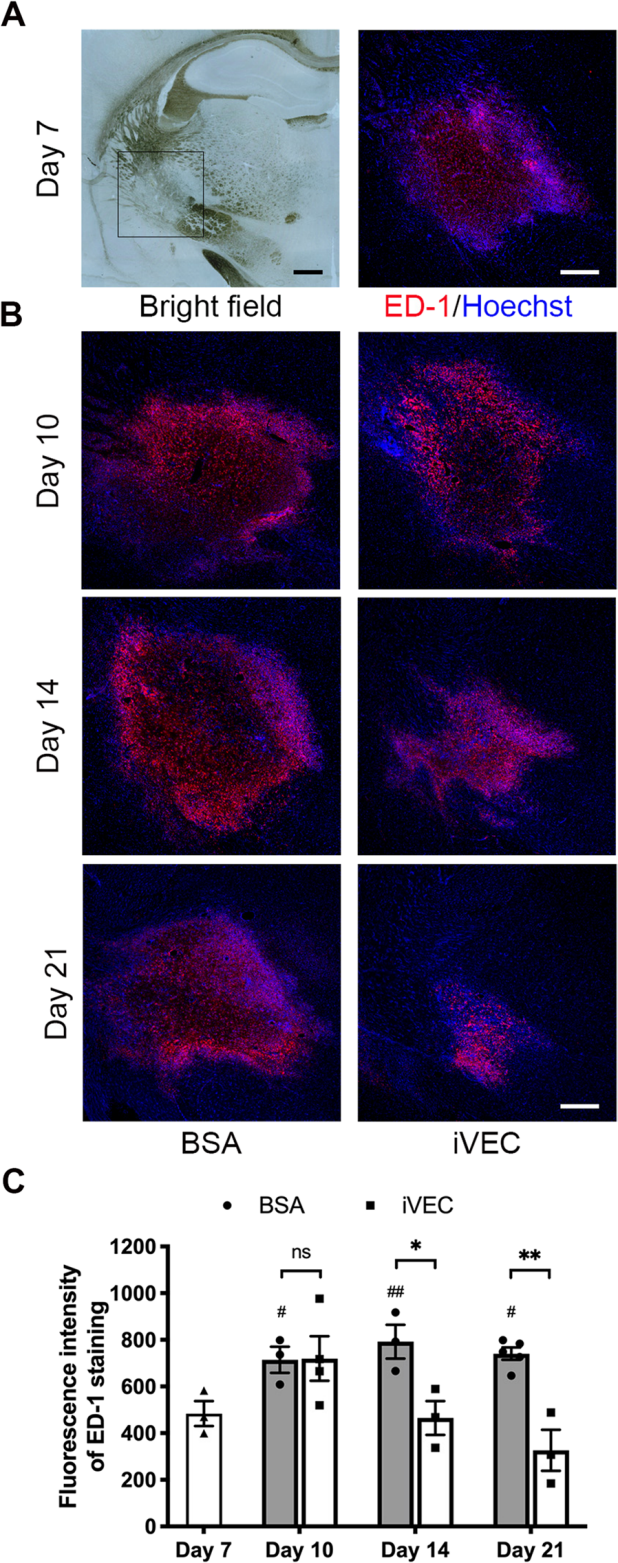
iVEC transplantation suppressed neuroinflammation in the ischemic white matter infarct. Immunohistochemistry with an ED-1 antibody was performed to detect activated microglia and macrophages in the infarct. Nuclei were stained by Hoechst 33342. **A** A typical bright field image of the white matter infarct on day 7 (left). Scale bar: 500 µm. An immunohistochemical image for ED-1 under higher magnification of the inset in “Bright field” (right). Scale bar: 200 µm. **B** Representative immunohistochemical images for ED-1 in iVEC-transplanted and BSA-injected brains at days 10, 14, and 21. Scale bar: 200 µm. **C** Quantification of fluorescence intensity of ED-1 staining in iVEC-transplanted and BSA-injected brains at days 10, 14, and 21. All data are expressed as mean ± SEM. *n* = 3–5 in each group. ^*^p < 0.05 and ^**^p < 0.01. ns, no significant difference. ^#^*p* < 0.05 and ^##^*p* < 0.01 compared to Day 7 group. These experiments were repeated three times, and similar results were obtained each time. Typical experiments are shown here

White matter infarct model was established by injecting ET-1 into the left IC of rat brain. Seven days after ET-1 injection, T2-weighted MR imaging was performed to estimate the infarct size and location. White matter infarction was confirmed at day 7 and the infarct size was not significantly different between each experimental group before cell transplantation or vehicle injection (Additional file 1: Fig. S1). Many ED-1^+^ activated microglia and macrophages were observed in the ischemic lesion on day 7 (Fig. 1A). On day 7, iVECs were transplanted into the ET-1-injured IC. We reported that iVECs showed the characteristics of vascular endothelial cells and maintained these characteristics for 2 weeks after transplantation [11]. The density of ED-1^+^ cells significantly increased in the infarct area at day 10 in both iVEC-transplanted and BSA-injected brains. At days 14 and 21, however, ED-1^+^ cells decreased in iVEC-transplanted brains, while their density remained almost the same in BSA-injected brains, resulting in a significant difference in the density of these inflammatory cells between iVEC-transplanted and BSA-injected brains (Fig. 1B, C). These results confirmed our previous conclusion that iVEC transplantation dramatically suppressed the inflammatory response in white matter infarct lesion [11].

### iVEC transplantation suppressed CD4^+^ T cell infiltration in ET-1 injured IC

**Fig. 2:**
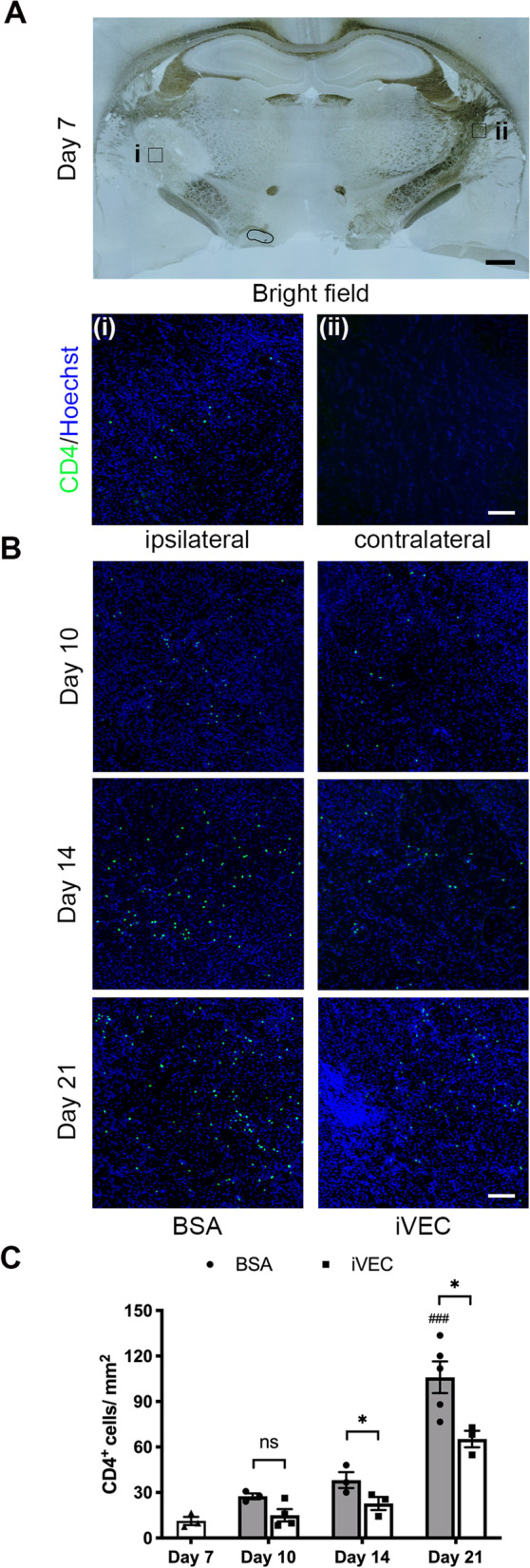
iVEC transplantation decreased CD4^+^ T cells in ischemic white matter infarct. Immunohistochemistry with a CD4 antibody was performed to quantify the number of CD4^+^ T cells. Nuclei were stained by Hoechst 33342. **A** A typical bright field image of the white matter infarct on day 7 (top). Scale bar: 500 µm. Immunohistochemical images for CD4 under higher magnification of the ipsilateral ischemic region (left) and the contralateral intact region (right). Scale bar: 50 µm. **B** Representative immunohistochemical images for CD4 in iVEC-transplanted and BSA-injected brains at days 10, 14, and 21. Scale bar: 50 µm. **C** Quantification of the CD4^+^ cell number in iVEC-transplanted and BSA-injected brains at days 10, 14, and 21. All data are expressed as mean ± SEM. *n* = 3–5 in each group. ^*^p < 0.05. ns, no significant difference. ^###^*p* < 0.001 compared to Day 7 group. These experiments were repeated three times, and similar results were obtained each time. Typical experiments are shown here

To evaluate whether T cells affect neuroinflammation in ischemic white matter damage, we immunohistochemically stained T cells using a CD4 antibody. CD4^+^ T cells were not observed in the intact contralateral white matter, while they were observed in ET-1 induced ischemic lesion on day 7 (Fig. 2A). CD4^+^ T cells increased gradually from day 7 to day 21 in BSA-injected rat brains (Fig. 2B, C). iVEC transplantation remarkably reduced the number of CD4^+^ T cells in ischemic IC compared to BSA-injected group on days 14 and 21 (Fig. 2B, C). These data indicated that transplanted iVECs suppressed increase of CD4^+^ T cells in the ischemic white matter lesion.

### iVEC transplantation increased the number of Tregs in ischemic infarct lesion

**Fig. 3:**
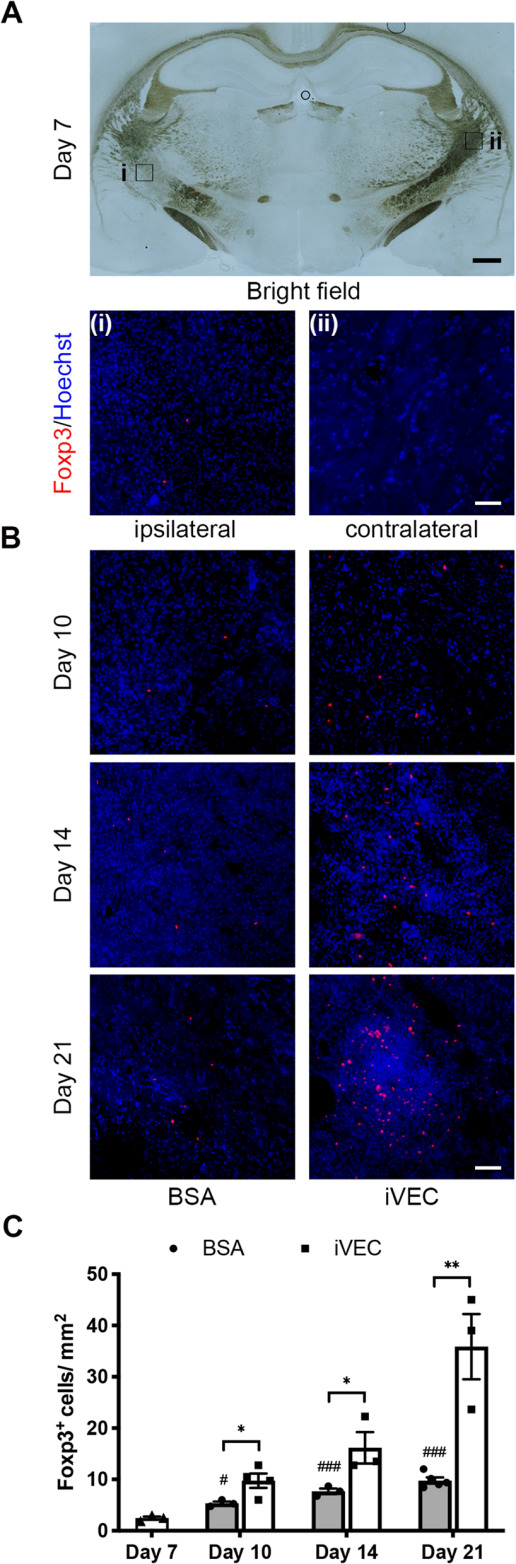
iVEC transplantation increased the Foxp3^+^ Tregs in ischemic white matter infarct. Immunohistochemistry with a Foxp3 antibody was performed to quantify the number of Foxp3^+^ Tregs. Nuclei were stained by Hoechst 33342. **A** A typical bright field image of the white matter infarct on day 7 (top). Scale bar: 500 µm. Immunohistochemical images for Foxp3 under higher magnification of the ipsilateral ischemic region (left) and the contralateral intact region (right). Scale bar: 50 µm. **B** Representative immunohistochemical images for Foxp3 in iVEC-transplanted and BSA-injected brains at days 10, 14, and 21. Scale bar: 50 µm. **C** Quantification of the Foxp3^+^ cell number in iVEC-transplanted and BSA-injected brains at days 10, 14, and 21. All data are expressed as mean ± SEM. *n* = 3–5 in each group. ^*^p < 0.05 and ^**^p < 0.01. ^#^*p* < 0.05 and ^###^*p* < 0.001 compared to Day 7 group. These experiments were repeated three times, and similar results were obtained each time. Typical experiments are shown here

Brain sections were stained by a Foxp3 antibody to examine the number of Tregs. While no Foxp3^+^ cells were observed in the intact contralateral region, a few Foxp3^+^ cells appeared in ischemic injured IC on day 7 (Fig. 3A), indicating that Foxp3^+^ Tregs were also involved in regulation of neuroinflammation in ET-1 induced white matter infarct lesion. Transplanted iVECs markedly increased Foxp3^+^ cells compared to BSA-injected group on days 10, 14 and 21 (Fig. 3B, C), indicating that iVEC transplantation promoted accumulation of Foxp3^+^ Tregs in white matter infarct region.

### Brain Tregs in ischemic white matter were recruited from the peripheral immune system by iVEC transplantation

**Fig. 4:**
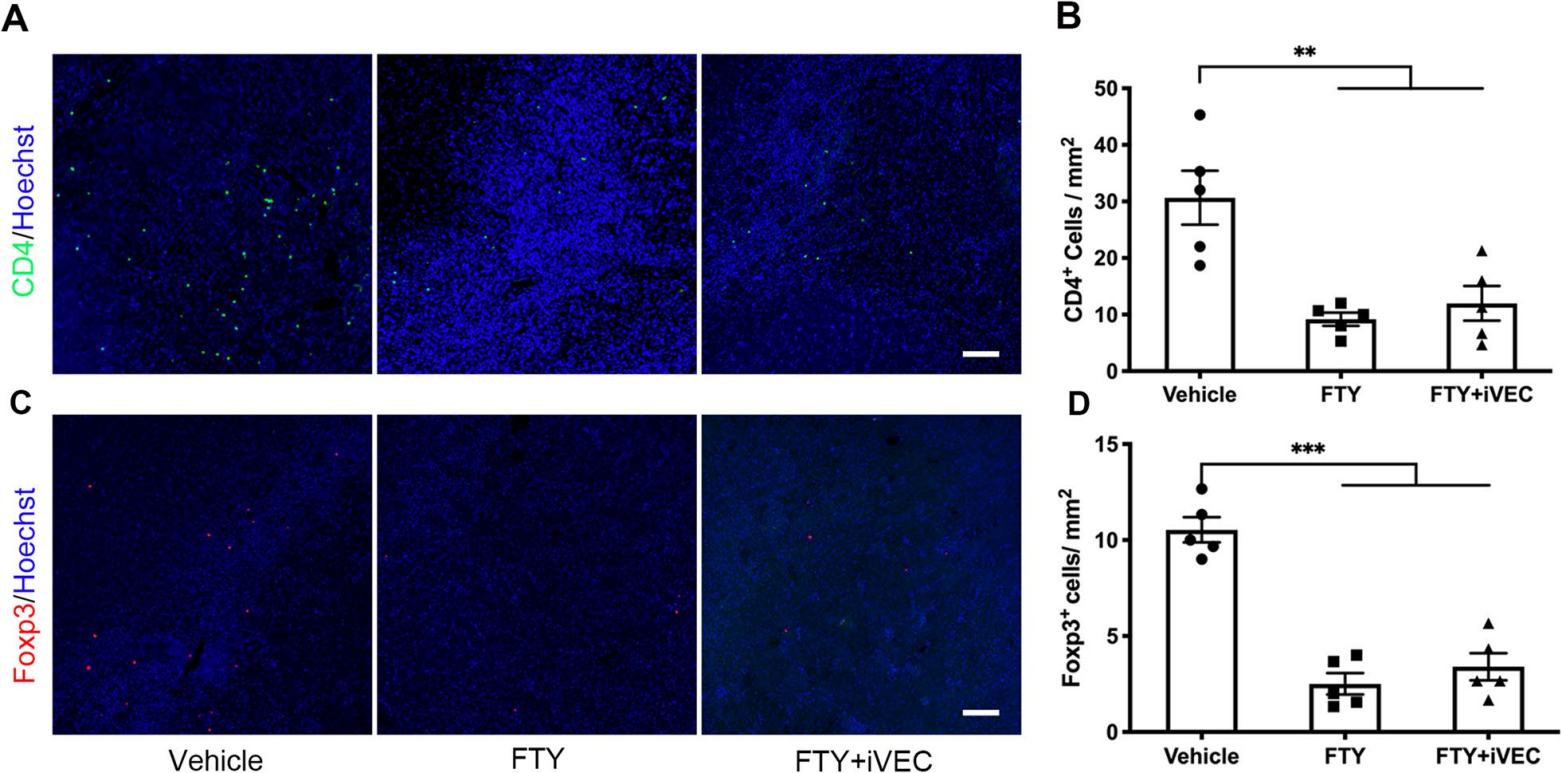
Sequestration of the immune cells in the periphery greatly reduced CD4^+^ T cells including Foxp3^+^ Tregs in the white matter infarct. Sequestration of the immune cells in the peripheral lymph nodes was performed by FTY720 treatment from days 7 to 14 after stroke onset. **A** Representative immunohistochemical images for CD4 in vehicle-injected (Vehicle), FTY720-treated (FTY), and FTY720-treated and iVEC-transplanted (FTY + iVEC) groups at day 14. Nuclei were stained by Hoechst 33,342. Scale bar: 50 µm. **B** Quantification of CD4^+^ T cell number in each group at day 14. All data are expressed as mean ± SEM. *n* = 5 in each group. ^**^p < 0.01. **C** Representative immunohistochemical images for Foxp3 in vehicle-injected (Vehicle), FTY720-treated (FTY), and FTY720-treated and iVEC-transplanted (FTY + iVEC) groups at day 14. Nuclei were stained by Hoechst 33342. Scale bar: 50 µm. **D** Quantification of Foxp3^+^ Treg number in each group at day 14. Note that the number of Foxp3^+^ Tregs in the FTY720-treated and iVEC-transplanted brain was significantly smaller than that of the vehicle-injected, FTY720-untreated brain, indicating that iVECs recruit Tregs from peripheral immune system to the ischemic infarct. All data are expressed as mean ± SEM. *n* = 5 in each group. ^***^p < 0.001. These experiments were repeated three times, and similar results were obtained each time. Typical experiments are shown here

To examine contribution of peripheral immune system to ET-1 induced white matter infarct, ET-1-injected rats were treated with FTY720, a sphingosine-1-phosphate receptor modulator, which sequesters lymphocytes in peripheral lymph nodes. One week after administration of FTY720 (day 14), the number of both brain CD4^+^ T cells and Tregs significantly decreased compared to vehicle-injected control (Fig. 4). These results indicate that both CD4^+^ T cells and Tregs originate from peripheral immune system. Moreover, when treated with FTY720 from days 7 to 14 after stroke onset, the number of Foxp3^+^ Tregs in the iVEC-transplanted brain was significantly smaller than that of vehicle-injected brain of the FTY720-untreated animal (Fig. 4C, D), indicating that iVECs recruit Tregs from peripheral immune system to the ischemic infarct.

### Sequestering immune cells in the periphery suppressed neuroinflammation, while it did not promote remyelination in the white matter infarct

**Fig. 5:**
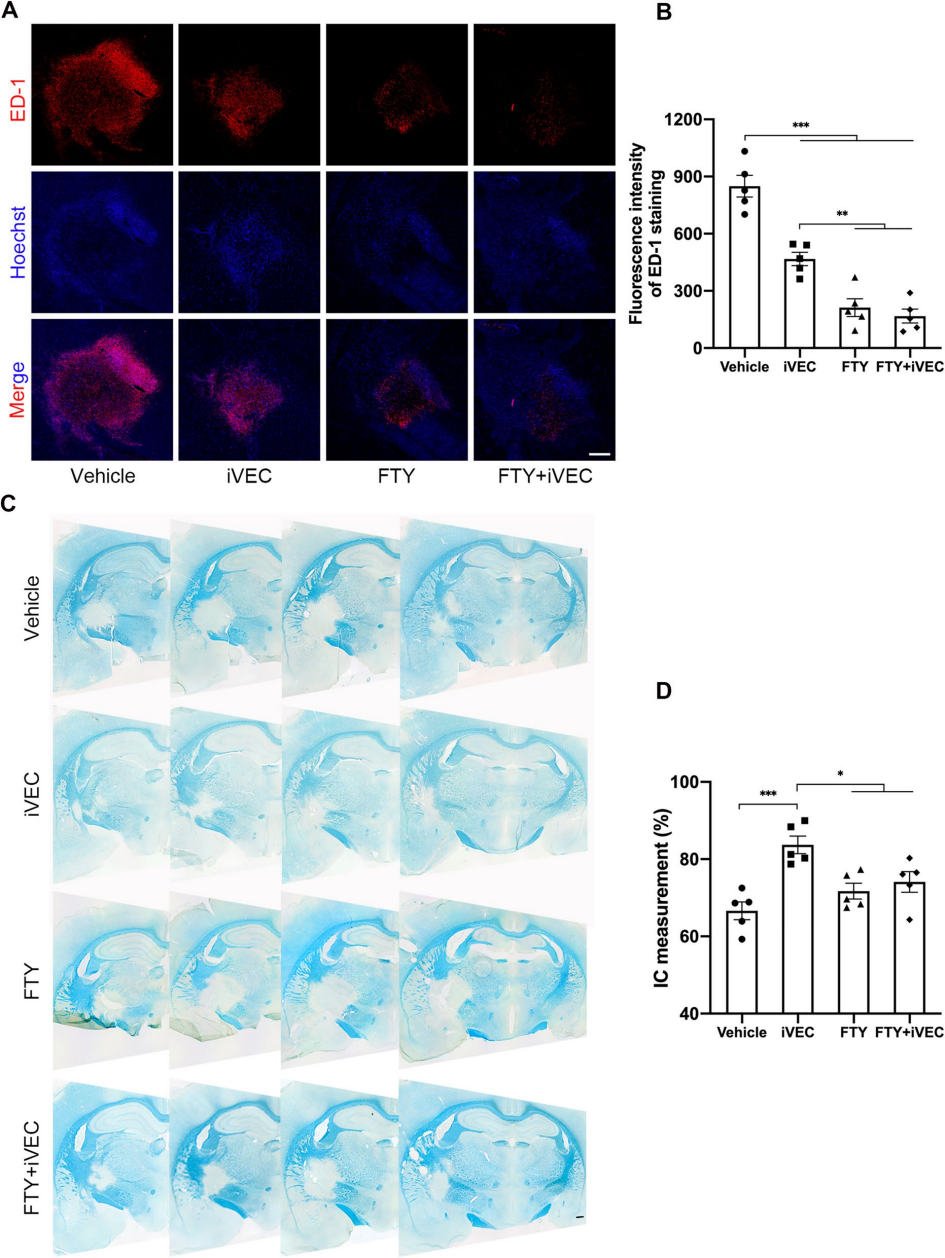
FTY720 treatment suppressed inflammatory response but did not promote recovery of the white matter infarct. FTY720 administration from days 7 to 14 after stroke onset was performed to inhibit the egress of T lymphocytes from lymph nodes.** A** Representative immunohistochemical images for ED-1 in vehicle-injected (Vehicle), iVEC-transplanted (iVEC), FTY720-treated (FTY), and FTY720-treated and iVEC-transplanted (FTY + iVEC) groups at day 14. Nuclei were stained by Hoechst 33342. Scale bar: 200 µm. **B** Quantification of fluorescence intensity of ED-1 staining in each group at day 14. All data are expressed as mean ± SEM. *n* = 5 in each group. ^**^p < 0.01 and ^***^p < 0.001. **C** Representative LFB staining images of rat brain sections in each group. Scale bar: 500 µm. **D** Quantification of the damaged IC volume in each group on day 14. All data are expressed as mean ± SEM. *n* = 5 in each group. ^*^p < 0.05 and ^***^p < 0.001. These experiments were repeated three times, and similar results were obtained each time. Typical experiments are shown here

As shown in Fig. 5A and B, FTY720 treatment from days 7 to 14 after stroke onset significantly reduced the number of ED-1^+^ inflammatory cells in the infarct area. FTY720 treatment had a stronger effect of inhibiting accumulation of inflammatory cells compared to iVEC transplantation. When remyelination was examined by LFB staining, however, FTY720 treatment did not promote recovery of the infarct area even in the presence of transplanted iVECs (Fig. 5C, D).

### iVEC transplantation increased the number of oligodendrocyte lineage cells without contribution of peripheral immune cells

**Fig. 6:**
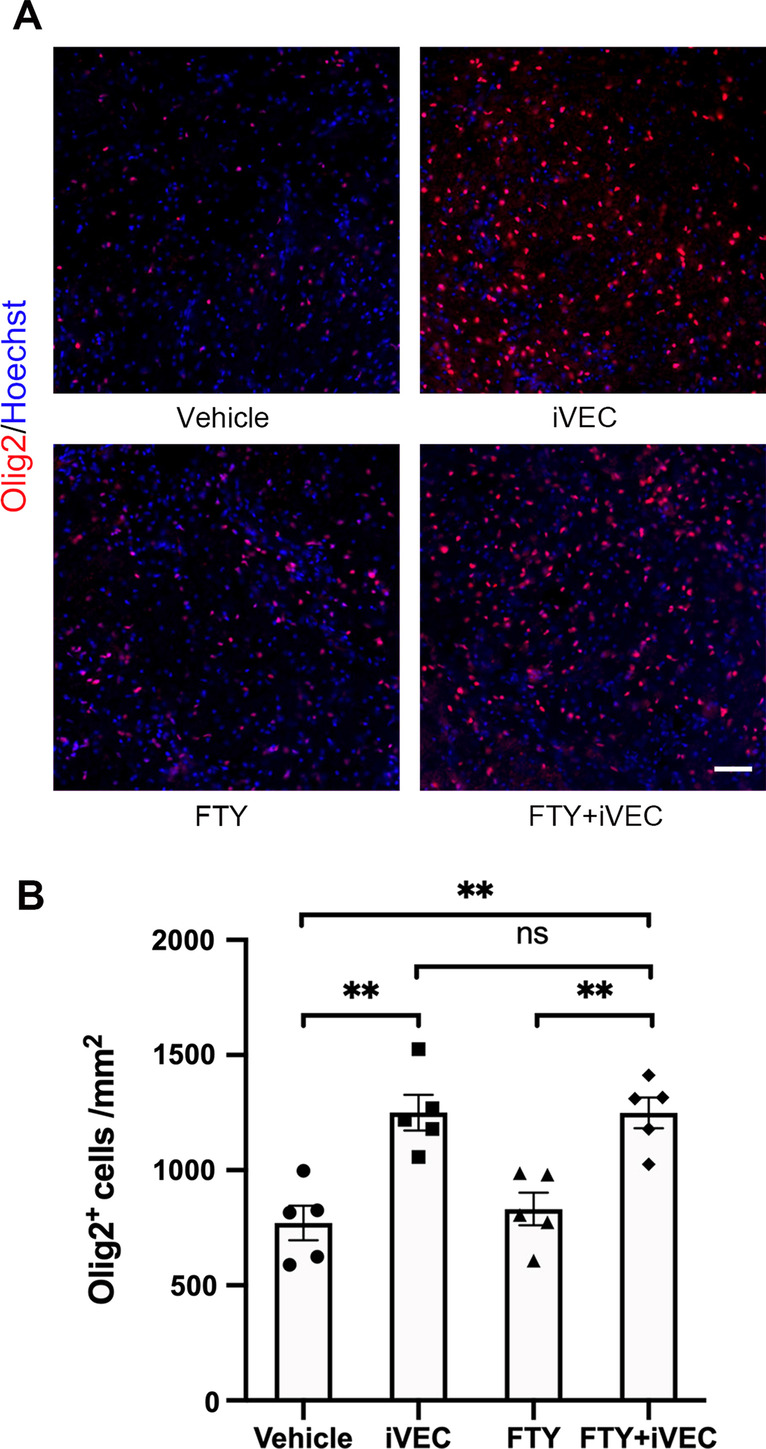
The increase of oligodendrocyte lineage cells by iVEC transplantation in the white matter infarct was not abrogated by FTY720 treatment. **A** Immunohistochemical images for Olig2 in vehicle-injected (Vehicle), iVEC-transplanted (iVEC), FTY720-treated (FTY), and FTY720-treated and iVEC-transplanted (FTY + iVEC) groups at day 14. Nuclei were stained by Hoechst 33342. Scale bar: 50 µm. **B** Quantification of Olig2^+^ cells in each group at day 14. All data are expressed as mean ± SEM. *n* = 5 in each group. ^**^p < 0.01. ns, no significant difference. These experiments were repeated three times, and similar results were obtained each time. Typical experiments are shown here

We previously showed that transplantation of vascular endothelial cells into the infarct area promotes increase of OPCs in the region [12]. To examine contribution of peripheral immune cells in this effect of vascular endothelial cell transplantation, egress of peripheral immune cells was inhibited by FTY720 treatment. As we previously reported, the number of Olig2^+^ oligodendrocyte lineage cells increased in ischemic area of iVEC-transplanted rats compared to vehicle-injected animals. FTY720 treatment had no effect, however, on the increase of Olig2^+^ cells by iVEC transplantation (Fig. 6A, B), indicating peripheral immune cells do not contribute to the increase of Olig2^+^ cells by iVEC transplantation.

### Transplantation of human Tregs together with iVECs into FTY720-treated animals promoted remyelination

**Fig. 7:**
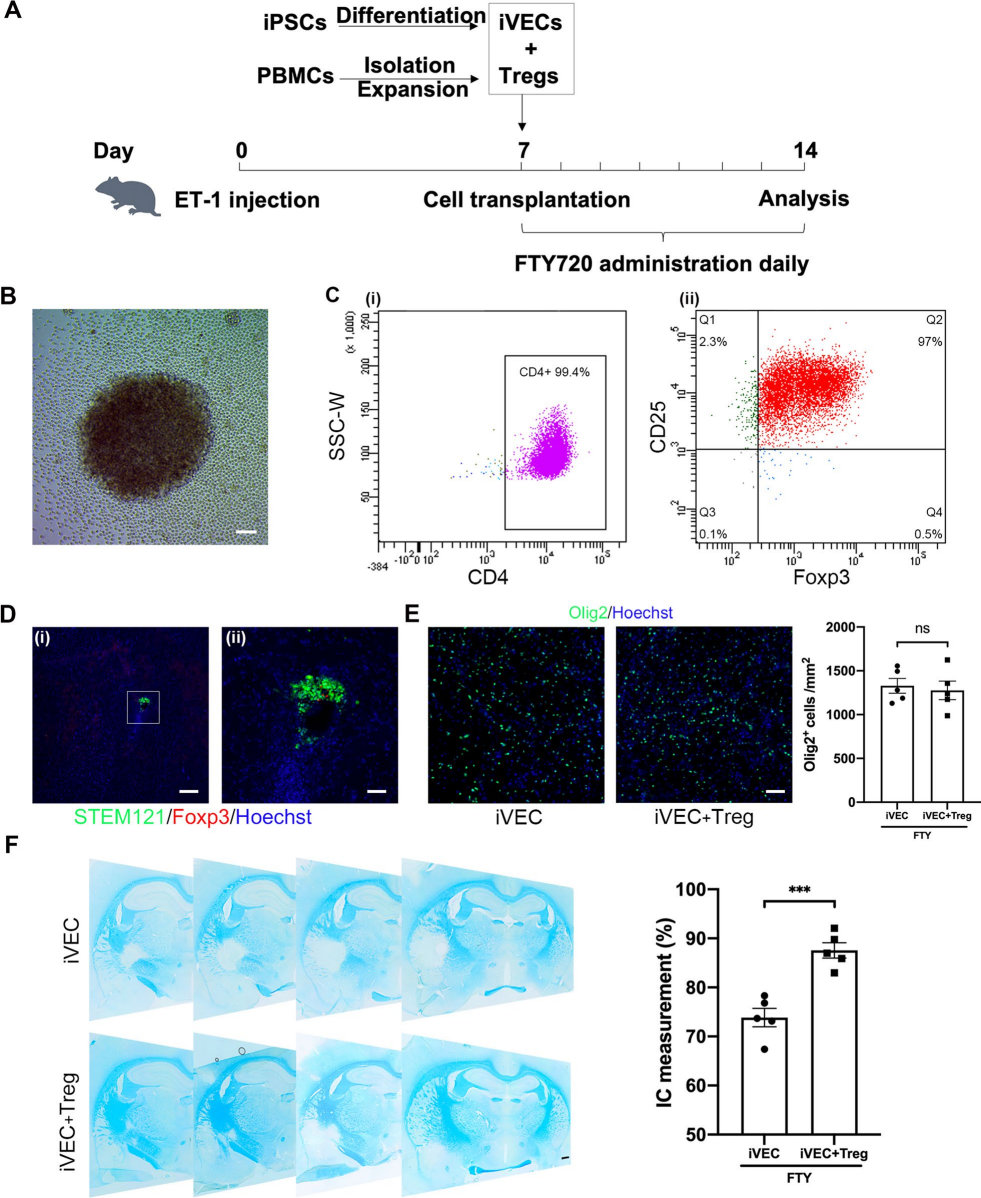
Transplantation of human Tregs together with iVECs into FTY720-treated animals promoted remyelination. **A** The timeline for the experimental procedure in this experiment is shown. Endothelin-1 was injected into the left IC to induce infarction on day 0. iVECs, Tregs isolated and expanded from human PBMCs, or bovine serum albumin (BSA) were injected into each animal on day 7. Some animals received intraperitoneal administration of FTY720 daily from day 7 to day 14. **B** A phase contrast image of cultured human Tregs on day 7. Scale bar: 200 µm. **C** FACS analysis for the marker expression of cultured human Tregs. (i) CD4^+^ T cells, (ii) CD25^+^Foxp3^+^ Tregs. **D** (i) Immunohistochemical images for STEM121 and Foxp3 in the iVEC- and Treg-transplanted brain section on day 14. (ii) Higher magnification image of the inset in (i). Nuclei were stained by Hoechst 33,342. Scale bars: 100 µm (i) and 50 µm (ii). **E** Immunohistochemical images for Olig2 and the quantification of Olig2^+^ cells in iVEC- and iVEC + Treg-transplanted animals treated with FTY720 on day 14. Nuclei were stained by Hoechst 33342. Scale bar: 50 µm. All data are expressed as mean ± SEM. *n* = 5 in each group. ns, no significant difference. **F** Representative LFB staining images of iVEC- and iVEC + Treg-transplanted brain sections and quantification of the damaged IC volume in each group on day 14. Scale bar: 500 µm. All data are expressed as mean ± SEM. *n* = 5 in each group. ^***^p < 0.001. These experiments were repeated three times using human Tregs obtained from three different donors, and similar results were obtained each time. Typical experiments are shown here

Sequestering immune cells in the periphery by FTY720 treatment suppressed neuroinflammation but did not promote remyelination (Fig. 5). On the other hand, treating animals with FTY720 greatly reduced the number of Foxp3^+^ Tregs in the infarct region (Fig. 4B). To examine the roles Tregs play in the recovery process of white matter infarct, iVECs were transplanted into the infarct region together with or without human Tregs 7 days after ET-1 injection (Fig. 7A). Tregs were isolated from human PBMCs by MACS technology. After stimulation with CD3/CD28 antibodies, these cells proliferated rapidly and showed a typical lymphocyte cluster in Treg culture medium containing human IL-2 (Fig. 7B). Fourteen days after expansion, flow cytometry analysis revealed that the expanded cells were Tregs, as most (approximately 94%) of these cells expressed CD4, CD25, and Foxp3 (Fig. 7C). These human Tregs were transplanted into the site of white matter infarct together with iVECs. Double immunohistochemistry for STEM121, a marker for human cytoplasm specific protein, and Foxp3 was performed to trace transplanted iVECs and human Tregs at day 14. Transplanted iVECs (STEM121^+^/ Foxp3^−^ cells) and human Tregs (STEM121^+^/ Foxp3^+^ cells) were found in ischemic brain section (Fig. 7D), indicating that these transplanted iVECs and human Tregs survived for at least one week after transplantation. The number of Olig2^+^ cells did not differ in iVEC-transplanted rats either in the absence or presence of human Tregs at day 14 (Fig. 7E), indicating that Tregs do not affect the number of oligodendrocyte lineage cells. LFB staining revealed, however, human Tregs significantly promoted the recovery of damaged IC (Fig. 7F).

## Discussion

We found that CD4^+^ T lymphocytes including Foxp3^+^ Tregs infiltrate into the ischemic demyelinating site from the periphery, as treatment of the animals with FTY720, a potent inhibitor of lymphocyte egress from the peripheral lymph nodes, remarkably reduced the number of these cells in the infarct region. FTY720 treatment strongly suppressed neuroinflammation in the infarct area, suggesting the importance of infiltration of peripheral immune cells to the infarct area in the aggravation of neuroinflammation. Although FTY720 treatment strongly suppressed neuroinflammation in the infarct area, it did not promote remyelination by itself, suggesting suppression of neuroinflammation per se cannot account for the beneficial effect of iVEC transplantation on white matter infarct. In accordance with this, we previously showed that iVEC transplantation increased the number of oligodendrocyte lineage cells [11].

Tregs, a subpopulation of CD4^+^ T lymphocytes, are essential for maintenance of peripheral immunological tolerance and prevention of autoimmunity, and have received extensive attention in the field of neuroinflammation in recent years. Brain Tregs suppress neuroinflammatory response, astrogliosis, and improve neurological recovery after ischemic stroke [16]. Treg deficiency aggravates injury of neurodegenerative disorders such as multiple sclerosis [20]. In the present study, we found appearance of Tregs in the infarct area on day 7 after induction of ischemic injury. Transplantation of iVECs on that day significantly increased the number of Tregs in the area thereafter, while the transplantation significantly reduced the number of total CD4^+^ T cells. The reason why iVEC transplantation reduces the number of total CD4^+^ cells while increasing the number of Tregs remains unsolved. There is a possibility that iVECs release chemotactic factors, such as chemokine ligand 1 (CCL1), CCL20, and CCL22 for Tregs. We are now examining this possibility. When human Tregs were transplanted together with iVECs into the white matter infarct region of the animals treated with FTY720, which had few endogenous Tregs in the lesions, remyelination was significantly promoted compared to the animals which received iVECs alone. This result clearly shows that Tregs per se promote remyelination in the infarct region. How Tregs promote remyelination remains undetermined. As transplanting human Tregs together with iVECs did not increase the number of oligodendrocyte lineage cells, Tregs apparently do not amplify oligodendrocyte lineage cells. As previously reported [21, 22], they may promote differentiation and maturation of oligodendrocyte lineage cells in the white matter of central nervous system. We are planning to examine this possibility in vitro and in vivo.

Weitbrecht and others reported that CD4^+^ T cells promote delayed B cell responses in the ischemic brain after stroke [23]. The responses of B cells after iVEC transplantation remain unclarified, and we will examine these in our next study.

Taken together, iVEC transplantation promotes remyelination in the white matter ischemic infarct via amplification of oligodendrocyte lineage cells, and recruitment of Tregs to the injured white matter, which probably promote differentiation and maturation of oligodendrocyte precursor cells.

## Conclusions

iVEC transplantation suppresses neuroinflammation, but suppression of neuroinflammation per se does not promote remyelination. Recruitment of Tregs by transplanted iVECs contributes significantly to promotion of remyelination in the injured white matter probably via promotion of differentiation and maturation of oligodendrocyte precursor cells.

## Supplementary Information

**Additional file 1: Fig. S1.** Establishment of the rat white matter infarct model. All animals received ET-1 injection into the left IC for induction of ischemic infarct on day 0. Half of the animals received BSA-injection and the other half received iVEC-transplantation on day 7. Some animals were treated with FTY720 from day 7 to day14. Infarct size was evaluated by MR imaging on days 7 and 14. **A** T2-weighted MR images of BSA-injected and iVEC-transplanted rat brain on day 7. Scar bar: 5 mm. **B** MR images were captured on days 7 and 14. Scar bar: 5 mm. **C** Quantification of the infarct area in ischemic brain at days 7 and 14. All data are expressed as mean ± SEM. *n* = 5 in each group. ns, no significant difference. These experiments were repeated three times, and similar results were obtained each time. Typical experiments are shown here.

## Data Availability

The data used and/or analyzed during the current study are available from the corresponding author on reasonable request.

## Abbreviations

BSA: Bovine serum albumin
CNS: Central nervous system
ET-1: Endothelin-1
EVs: Extracellular vesicles
FTY720: Fingolimod
HBSS: Hank’s balanced salt solution
hESFM: Human endothelial serum-free medium
IC: Internal capsule
iPSC: Induced pluripotent stem cell
iVEC: IPSC-derived vascular endothelial cell
LFB: Luxol fast blue
MACS: Magnetic-activated cell sorting
MR: Magnetic resonance
OPC: Oligodendrocyte precursor cell
PBMC: Peripheral blood mononuclear cell
PBS: Phosphate-buffered saline
PDS: Platelet-poor human plasma derived serum
Treg: Regulatory T cell
UM: Unconditioned medium

## References

[1] Mestre H, Du T, Sweeney AM, Liu G, Samson AJ, Peng W, Mortensen KN, Stæger FF, Bork PAR, Bashford L (2020). Cerebrospinal fluid influx drives acute ischemic tissue swelling. Science.

[2] Pandian JD, Gall SL, Kate MP, Silva GS, Akinyemi RO, Ovbiagele BI, Lavados PM, Gandhi DBC, Thrift AG (2018). Prevention of stroke: a global perspective. Lancet.

[3] Hong P, Gu R-N, Li F-X, Xiong X-X, Liang W-B, You Z-J, Zhang H-F (2019). NLRP3 inflammasome as a potential treatment in ischemic stroke concomitant with diabetes. J Neuroinflammation.

[4] Stoll G, Nieswandt B (2019). Thrombo-inflammation in acute ischaemic stroke - implications for treatment. Nat Rev Neurol.

[5] Li S, Rao JH, Lan XY, Li X, Chu CY, Liang Y, Janowski M, Zhang HT, Walczak P (2021). White matter demyelination predates axonal injury after ischemic stroke in cynomolgus monkeys. Exp Neurol.

[6] Al Mamun A, Chauhan A, Qi S, Ngwa C, Xu Y, Sharmeen R, Hazen AL, Li J, Aronowski JA, McCullough LD, Liu F (2020). Microglial IRF5-IRF4 regulatory axis regulates neuroinflammation after cerebral ischemia and impacts stroke outcomes. Proc Natl Acad Sci U S A.

[7] Lemarchant S, Dunghana H, Pomeshchik Y, Leinonen H, Kolosowska N, Korhonen P, Kanninen KM, Garcia-Berrocoso T, Montaner J, Malm T, Koistinaho J (2016). Anti-inflammatory effects of ADAMTS-4 in a mouse model of ischemic stroke. Glia.

[8] Song S, Wang S, Pigott VM, Jiang T, Foley LM, Mishra A, Nayak R, Zhu W, Begum G, Shi Y (2018). Selective role of Na(+) /H(+) exchanger in Cx3cr1(+) microglial activation, white matter demyelination, and post-stroke function recovery. Glia.

[9] Qin C, Fan WH, Liu Q, Shang K, Murugan M, Wu LJ, Wang W, Tian DS (2017). Fingolimod protects against ischemic white matter damage by modulating microglia toward M2 polarization via STAT3 pathway. Stroke.

[10] Puentes S, Kurachi M, Shibasaki K, Naruse M, Yoshimoto Y, Mikuni M, Imai H, Ishizaki Y (2012). Brain microvascular endothelial cell transplantation ameliorates ischemic white matter damage. Brain Res.

[11] Xu B, Kurachi M, Shimauchi-Ohtaki H, Yoshimoto Y, Ishizaki Y (2020). Transplantation of iPS-derived vascular endothelial cells improves white matter ischemic damage. J Neurochem.

[12] Iijima K, Kurachi M, Shibasaki K, Naruse M, Puentes S, Imai H, Yoshimoto Y, Mikuni M, Ishizaki Y (2015). Transplanted microvascular endothelial cells promote oligodendrocyte precursor cell survival in ischemic demyelinating lesions. J Neurochem.

[13] Kurachi M, Mikuni M, Ishizaki Y (2016). Extracellular vesicles from vascular endothelial cells promote survival, proliferation and motility of oligodendrocyte precursor cells. PLoS ONE.

[14] Osawa S, Kurachi M, Yamamoto H, Yoshimoto Y, Ishizaki Y (2017). Fibronectin on extracellular vesicles from microvascular endothelial cells is involved in the vesicle uptake into oligodendrocyte precursor cells. Biochem Biophys Res Commun.

[15] Stevens SL, Bao J, Hollis J, Lessov NS, Clark WM, Stenzel-Poore MP (2002). The use of flow cytometry to evaluate temporal changes in inflammatory cells following focal cerebral ischemia in mice. Brain Res.

[16] Ito M, Komai K, Mise-Omata S, Iizuka-Koga M, Noguchi Y, Kondo T, Sakai R, Matsuo K, Nakayama T, Yoshie O (2019). Brain regulatory T cells suppress astrogliosis and potentiate neurological recovery. Nature.

[17] Zhang D, Ren J, Luo Y, He Q, Zhao R, Chang J, Yang Y, Guo ZN (2021). T cell response in ischemic stroke: from mechanisms to translational insights. Front Immunol.

[18] Zhang Y, Liesz A, Li P (2021). Coming to the rescue: regulatory T cells for promoting recovery after ischemic stroke. Stroke.

[19] Ono H, Imai H, Miyawaki S, Nakatomi H, Saito N (2016). Rat white matter injury model induced by endothelin-1 injection: technical modification and pathological evaluation. Acta Neurobiol Exp (Wars).

[20] Jones A, Hawiger D (2017). Peripherally induced regulatory T cells: recruited protectors of the central nervous system against autoimmune neuroinflammation. Front Immunol.

[21] Dombrowski Y, O'Hagan T, Dittmer M, Penalva R, Mayoral SR, Bankhead P, Fleville S, Eleftheriadis G, Zhao C, Naughton M (2017). Regulatory T cells promote myelin regeneration in the central nervous system. Nat Neurosci.

[22] McIntyre LL, Greilach SA, Othy S, Sears-Kraxberger I, Wi B, Ayala-Angulo J, Vu E, Pham Q, Silva J, Dang K (2020). Regulatory T cells promote remyelination in the murine experimental autoimmune encephalomyelitis model of multiple sclerosis following human neural stem cell transplant. Neurobiol Dis.

[23] Weitbrecht L, Berchtold D, Zhang T, Jagdmann S, Dames C, Winek K, Meisel C, Meisel A (2021). CD4(+) T cells promote delayed B cell responses in the ischemic brain after experimental stroke. Brain Behav Immun.

