# Supplementary figures and images for: Transplanted human iPSC-derived vascular endothelial cells promote functional recovery by recruitment of regulatory T cells to ischemic white matter in the brain
Source: J Neuroinflammation. 2023 Jan 17;20:11. doi: 10.1186/s12974-023-02694-0 (PMC9847196; doi:10.1186/s12974-023-02694-0)

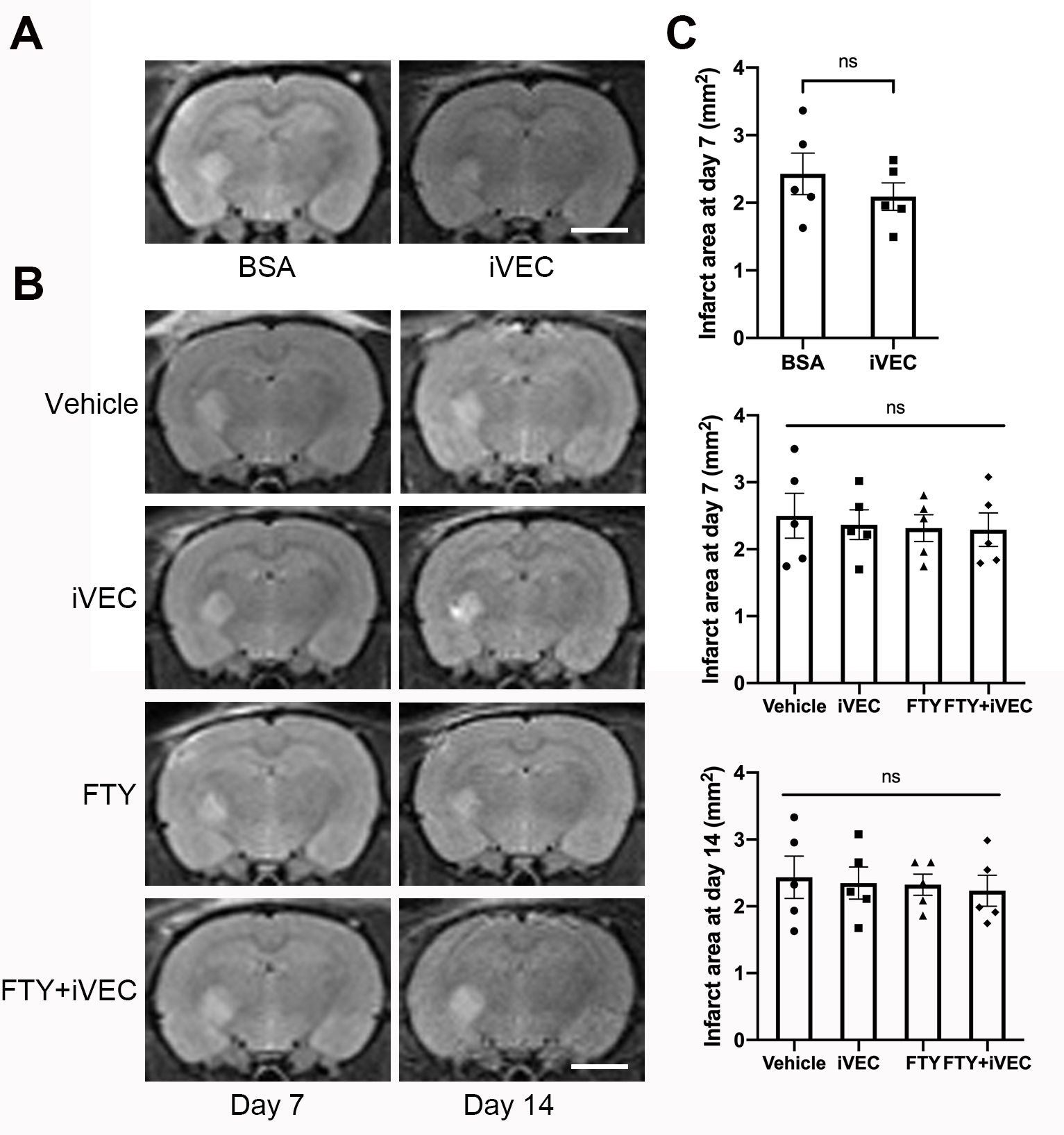

Supplement: Supplementary file 1 — Additional file 1: Fig. S1. Establishment of the rat white matter infarct model. All animals received ET-1 injection into the left IC for induction of ischemic infarct on day 0. Half of the animals received BSA-injection and the other half received iVEC-transplantation on day 7. Some animals were treated with FTY720 from day 7 to day14. Infarct size was evaluated by MR imaging on days 7 and 14. A T2-weighted MR images of BSA-injected and iVEC-transplanted rat brain on day 7. Scar bar: 5 mm. B MR images were captured on days 7 and 14. Scar bar: 5 mm. C Quantification of the infarct area in ischemic brain at days 7 and 14. All data are expressed as mean ± SEM. n = 5 in each group. ns, no significant difference. These experiments were repeated three times, and similar results were obtained each time. Typical experiments are shown here. [file 12974_2023_2694_MOESM1_ESM.jpg]
